# Scalable Genome Assembly through Parallel *de Bruijn* Graph Construction for Multiple *k*-mers

**DOI:** 10.1038/s41598-019-51284-9

**Published:** 2019-10-16

**Authors:** Kanak Mahadik, Christopher Wright, Milind Kulkarni, Saurabh Bagchi, Somali Chaterji

**Affiliations:** 1 Adobe Research, San Jose, USA; 2 Purdue University, West Lafayette, IN USA

**Keywords:** Computational models, Computational platforms and environments

## Abstract

Remarkable advancements in high-throughput gene sequencing technologies have led to an exponential growth in the number of sequenced genomes. However, unavailability of highly parallel and scalable *de novo* assembly algorithms have hindered biologists attempting to swiftly assemble high-quality complex genomes. Popular *de Bruijn* graph assemblers, such as IDBA-UD, generate high-quality assemblies by iterating over a set of *k*-values used in the construction of de Bruijn graphs (DBG). However, this process of *sequentially* iterating from small to large *k*-values slows down the process of assembly. In this paper, we propose ScalaDBG, which metamorphoses this sequential process, building DBGs for each distinct *k*-value in parallel. We develop an innovative mechanism to “patch” a higher *k*-valued graph with contigs generated from a lower *k*-valued graph. Moreover, ScalaDBG leverages multi-level parallelism, by both scaling up on all cores of a node, and scaling out to multiple nodes *simultaneously*. We demonstrate that ScalaDBG completes assembling the genome faster than IDBA-UD, but with similar accuracy on a variety of datasets (6.8X faster for one of the most complex genome in our dataset).

## Introduction

A principal component of computational genomics is sequence assembly, constructing the original genome sequence by combining *reads*, or fragments thereof, obtained from sequencing machines. *De novo* assembly is a sequence assembly technique that does not use a reference genome during reconstruction, and hence can facilitate the biological understanding of new or uncharacterized species. The process of mapping the sequenced reads for *de novo* assembly is complicated because of the lack of a reference sequence to which the sequenced reads can be aligned. Factors such as massive read sets, distinct error profiles introduced by sequencing machines, repeats in the original genome, and uneven sampling of the reference genome make this process computationally intensive. These exacting factors are exacerbated in metagenomics and single-cell sequencing datasets in tandem with the explosive growth in genomics data—2^40^ exabytes by 2025, just by taking into account human genomes^1^. This “genomical” data race has created an urgent need to speed up *de novo* assembly algorithms.

Popular assemblers such as Velvet^2^, ABySS^3^, and ALLPATHS-LG^4^ use a de Bruijn Graph (DBG)^5^ to perform *de novo* assembly. A DBG is a directed graph whose vertices are *k*-mers, or length-*k* substrings of the reads. An edge exists between two vertices if they are consecutive *k*-mers in a read and they share an overlap of a (*k* − 1)-mer^6^. To obtain contigs, or long contiguous genomic sequences, the DBG is traversed to identify *maximal paths i*.*e*., paths in which all vertices have an in-degree and out-degree equal to 1, except for the terminal vertices. These contigs are further assembled into longer regions or *scaffolds* based on their relative order and orientation.

The *k*-value chosen for DBG construction influences its structure. A small *k*-value cannot distinguish repeats or duplication due to erroneous reads (of length greater than *k*), and connects *k*-mers with other (false-positive) *k*-mers. This results in a *branched DBG*, with vertices having out-degree higher than 1, and terminating maximal paths, resulting in smaller-sized contigs. A large *k*-value, on the other hand, can differentiate among smaller repeats (of length less than *k*), and hence, reduces the number of branches. However, due to low or non-uniform sampling, some *k*-mers that introduce vertices and edges in the DBG are missed, resulting in a *fragmented DBG with dead*-*end paths*. This occurs if reads covering consecutive *k*-mers are missing, with increasing *k*-values exacerbating the fragmentation problem. Thus, selecting the correct value for the *k* parameter in DBG algorithms is crucial. Striking the correct balance between the *branching* and *fragmentation* problems is key to high assembly performance.

Based on this insight, several assemblers such as IDBA (Iterative DBG Assembler)^6^, IDBA-UD^7^, SOAPdenovo2^8^ and SPAdes^9^ use several different *k*-values during assembly. Intuitively, contigs from a smaller *k*-valued graph can be used to “patch up” gaps in the larger *k*-valued graph, while contigs from the larger *k*-valued graph can be used to resolve “branches” or conflicts in the smaller *k*-valued graph. SPAdes follows an iterative graph construction process using multiple *k*-values to construct a multi-sized DBG. IDBA iterates from small to large values of *k*, maintaining an accumulated DBG to carry useful information forward as it moves on to higher *k*-values. These iterative approaches establish that DBGs built with multiple *k*-values generate finer-quality assemblies than a DBG built with a single (be it large or small) *k*-value^6–9^. IDBA-UD is an improved version of IDBA, and *in the rest of the paper*, *we only refer to IDBA*-*UD for our experiments*.

## Motivation for ScalaDBG

**Figure 1:**
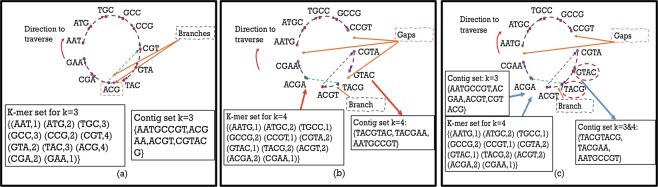
Original Genome Sequence: **AATGCCGTACGTACGAA**, Input Reads: *AATGC*, *ATGCC*, *GCCGT*, *TGCCG*, *CGTAC*, *TACGT*, *ACGTA*, *TACGA*, *ACGAA*. Figure shows the effect of using a small *k* ($k=3$) and a larger *k* ($k=4$) during DBG construction. Sub-figure (**a**) shows the graph constructed from read set with $k=3$. The vertices are 3-mers of the read set. They are connected to each other if they have a 2-mer overlap *and* if they are consecutive 3-mers in a read. This graph has branching at vertex *ACG* due to a repeating region in the genome *ACGT* and *ACGA*. The contig set generated by identifying maximal paths in the graph is *{AATGCCGT*, *ACGAA*, *ACGT*, *CGTACG}*. As the value of *k* is increased to 4 (sub-figure (**b**)), the branch disappears as the higher *k*-value can now distinguish between the repeat region in *ACGT* and *ACGA*. However, some reads such as *CCGTA* and *GTACG* are not sampled from a contiguous genome sequence and so vertices and edges in the graph are missed. For e.g., *GTAC* and *TACG* cannot be connected. While they do share a 3-mer overlap, they are not part of the same read. If the read *GTACG* would have been part of the read set, we could have connected them. The final graph (sub-figure (**c**)) can be created by filling in some of the gaps in the $k=4$ graph with contigs from the $k=3$ graph. The vertices for which new edge is added (sub-figure (**a**)) are circled. The final contig set corresponds to contigs in this graph.

While leveraging multiple *k*-values during the assembly improves its quality, the time taken to perform the assembly process also increases significantly. As described in Table 1 of our previous work^10^, the total time taken by IDBA-UD to assemble a medium-complexity CAMI metagenomics dataset increases linearly in proportion to the number of *k*-values used. Furthermore, among the different stages in IDBA-UD assembly, reading the sequence file (**Stage 1**), processing with multiple *k*-values (first, building the graph, and then iterating over the graph with several different *k*-values (**Stage 2**)), and finally scaffolding (**Stage 3**) to get the final assembly, the iterative graph construction process contributes to 96.1% of the total execution time, as shown in Fig. 1 of our previous work^10^. Notably, the graph-construction step (**Stage 2**), consisting of building an accumulated DBG by iterating over several different *k*-values, is the bottleneck in the assembly workflow.

**Table 1:** Read Sets used in the Experiments.

Name	Read Set Type	Read Length	# of Reads	Characteristics
RM1	Real, Metagenomic	150 bp	33140480	PE, Insert size:5 kbp
RM2	Real, Metagenomic	150 bp	33128228	PE, Insert size:5 kbp
SC-*E*. *coli*	Real, Single Cell	100 bp	23,818,596	PE, Insert size:266 bp
SC-*S*. *aureus*	Real, Single Cell	100 bp	66,997,488	PE, Insert size:214 bp
SC-SAR324	Real, Single Cell	100 bp	55,733,218	PE, Insert size:180 bp

PE denotes Paired-end reads.

## Our ScalaDBG System

To address this concern, we propose *ScalaDBG*, a new parallel assembly algorithm that parallelizes Stage 2 of the assembly workflow, the iterative DBG construction process with multiple *k*-values. The key insight behind ScalaDBG is that the graphs for multiple *k*-values need not be constructed serially. Instead, *each graph construction can be done independently and in parallel*. Accumulating the graph for the higher *k*-value, such as in IDBA-UD, introduces an apparent dependency on the graph with lower *k*-values. We remove this dependency, by introducing a *patching technique*, which can patch the higher *k*-valued graph (*k*_2_) with contigs from the lower *k*-valued graph (*k*_1_). Crucially, the first stage of graph construction of the *k*_1_ and *k*_2_ graphs can proceed in parallel and the relatively shorter stage of patching the *k*_2_ graph with the contigs from the smaller *k*-valued, *k*_1_ graph, happens subsequently. Thus, the more fragmented, higher *k*-valued graphs are *cemented* from the contigs of the lower *k*-valued graphs, with the branches of the lower *k*-valued graph being simultaneously removed.

ScalaDBG first performs graph construction in parallel for each *k*-value. Next, for each pair of graphs, the higher *k*-valued graph is patched using the lower *k*-valued graph to generate a single graph. Note that there are several independent patch processes, for a long chain of *k*-values, and they execute in parallel. This process is performed recurrently, until there is only a single graph, similar to a *parallel tree*-*reduction* model. Thus ScalaDBG breaks a sequentially executed chain of *k*-values, executing both construction and patching in parallel, with higher parallelism for a longer chain of *k*-values. *ScalaDBG is the first assembler to parallelize multi*-*k*-*valued DBG construction*. We show in our evaluation that there is no statistical difference in the assembly quality of ScalaDBG and IDBA-UD.

## Removing the Assembly Kernel’s Time-Complexity Bottleneck in Genome Analyses Pipelines Using ScalaDBG

Genome analysis pipelines have several repetitive “kernels” or algorithmic modules as we have seen in the process of building our own domain-specific language (DSL) for genomics^11^. In a typical genome analysis pipeline, generation of genomics reads from sequencing machines is followed by the kernel(s) of genome assembly, and subsequently, analysis of the assembled sequences using a repertoire of metrics. Tremendous improvements in sequencing machines have made the genome assembly kernel the bottleneck in the process of extracting meaningful insights from raw genomics datasets. A high-latency genome assembly kernel negatively affects the subsequent analysis kernels and overall pipeline performance, especially in high-use genomics pipelines, such as MG-RAST^12,13^, reducing the throughput of production-scale high-performance computing pipelines^14,15^. ScalaDBG can be deployed on modest hardware *e*.*g*., multiple nodes in a supercomputer or commercially available cloud infrastructure (*e*.*g*., AWS Amazon cloud, Google cloud) to speed up assembly for a much lower cost. In contrast, the scalability of IDBA-UD is severely limited by the memory and compute capacity of server nodes, which are expensive to upgrade.

## Multi-Grained Parallelism of ScalaDBG

ScalaDBG leverages parallelism at two levels—first: constructing several different *k*-valued graphs in parallel, and second: parallelizing processes such as *k*-mer counting, indexing, and lookup that occur within each graph-construction step. This strategy of ScalaDBG enables us to leverage the *hybrid MPI*-*Open MP parallel programming model*. While each MPI process can independently perform graph construction on different nodes in a cluster, Open-MP threads can exploit all cores on a single node. Thus, ScalaDBG utilizes both *vertical scaling or scaleup* in a powerful server node with a higher core-count, *and horizontal scaling or scale*-*out* in a cluster with multiple nodes. ScalaDBG completes the assembly of a SAR metagenomic dataset with a set of *k*-values in the range of 20–50, with a step size of 2, 6.8X faster than IDBA-UD, reducing the execution time from ~2 hours to 17 minutes.

We make the following technical contributions in this paper:

1. We break the dependency in DBG creation for multiple *k*-values—from a purely serial process to one where the most time-consuming part (the DBG creation for individual *k*-values) is parallelized. This innovation can be applied out-of-the-box to most DBG-based assemblers.
2. We develop a divide-and-conquer strategy for handling a long sequential chain of *k*-values, which improves the quality of assembly
3. We develop a software package called ScalaDBG that uses Open MP for the scale-up operation within one server and MPI for the scale-out process across multiple servers. The software package is available at https://github.com/purdue-dcsl/Scaladbg.

## Related Work

Several effective *de*-*novo* assembly applications have been put forward to deal with the deluge in genomic sequences^2–4,6–9,16–21^. However, these applications are restricted to scaling up on a multi-core machine, or do not use several *k*-values during assembly. To the best of our knowledge, there has been no prior work on distributed and parallelized DBG construction for multiple *k*-values.

In previous work, Ray^16^, ABySS^3^, PASHA^17^, and HipMer^18^ can distribute the task of DBG construction to different nodes in a cluster. Metagenomics assemblers, such as Meta-velvet^19^ also do not apply multiple *k*-values. However, this approach performs poorly for datasets with uneven sequencing depths, such as in metagenomics and single-cell datasets. ScalaDBG employs multiple *k*-values to deal with such datasets. On the other hand, SGA^21^, Velvet^2^, SOAPdenovo^8^, ALLPATHS-LG^4^ are limited to scaling up on a multi-core node. Additionally, while IDBA, IDBA-UD, and SPAdes can operate on several *k*-values, their scaling is restricted to multiple cores on a single node. In contrast, ScalaDBG is a distributed and parallelized assembler operating on multiple *k*-values.

### IDBA-UD as our algorithm for benchmarking

IDBA-UD is a an iterative *k*-value DBG-based assembler that runs through a range of *k* values from $k={k}_{min}$ to $k={k}_{max}$, with a step-wise increment of *s*. It maintains an accumulated DBG *H*_*k*_ at each step. In the first step, a DBG ${G}_{{k}_{min}}$ is generated from the input reads. For $k={k}_{min}$, *H*_*k*_ is equivalent to ${G}_{{k}_{min}}$. After DBG construction, contigs for graph *H*_*k*_ are generated by considering all maximal paths in graph *H*_*k*_. All vertices in any maximal path have an in-degree and out-degree equal to 1, except the vertices at the start and end of the path. Subsequently, reads from the input set that are substrings of these contigs are removed. This generally reduces the size of the input read set. Note that, a read of length *r* generates $r-k+1$ vertices. Thus, as *k* is increased, each read introduces fewer vertices. This reduction in size of the input read set, coupled with the fact that there are fewer vertices for larger *k*-values, makes subsequent graph-construction steps less time consuming. For the next iteration, where $k={k}_{min}+s$, the graph *H*_*k*_, the remaining reads, and the contigs from *H*_*k*_ are fed as inputs. Every *s*-length path in *H*_*k*_ is upgraded to a vertex. A $(k+s+1)$-mer in either the remaining reads or the contigs of *H*_*k*_ is used to connect vertices in *H*_*k*_. The next set of iterations continue this process until $k={k}_{max}$ is reached. Observe that in this algorithm, at iteration *i*, graph ${H}_{{k}_{min}+i\ast s}$ depends on graph ${H}_{{k}_{min}+(i-1)\ast s}$, the reduced read set, and the contigs obtained at the previous iteration (*i* − 1). *This dependency compels IDBA*-*UD to work sequentially on the chain of k*-*values*, *irrespective of the length of the chain*. This is the essence of the problem that we tackle in ScalaDBG.

## Methods - Design of ScalaDBG

Our technique, ScalaDBG, consists of two phases—the **build phase** (building a DBG) and the **patch phase** (patching a partial DBG with contigs from a lower *k*-valued DBG). In addition, it employs an efficient scheduler to fully exploit all nodes in a cluster.

### Build phase

**Figure 2:**
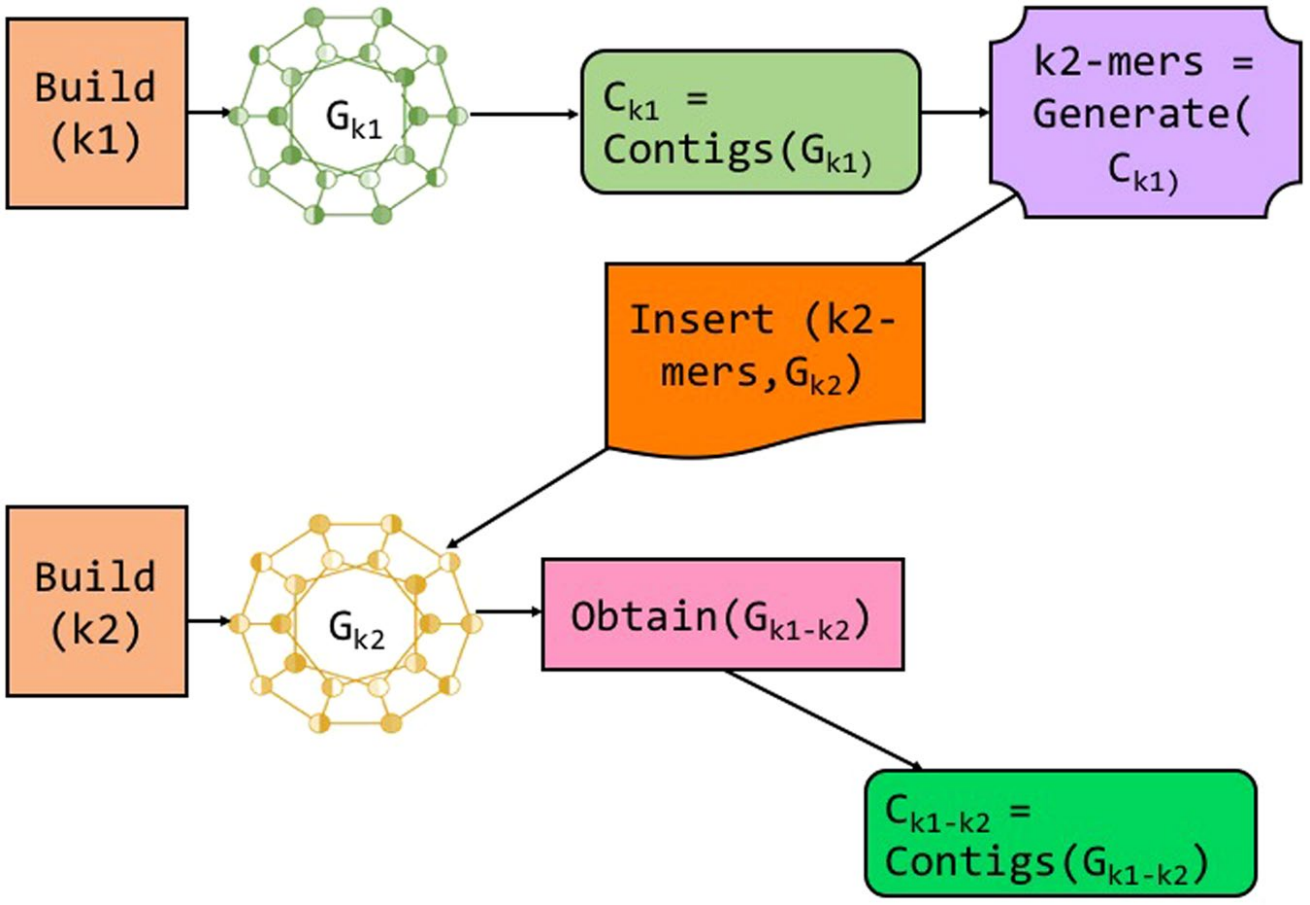
High Level Architecture Diagram of ScalaDBG. This shows the graph construction with only two different *k* values, *k*1 and *k*2 with $k1 < k2$. The graph *G*_*k*2_ is “patched” with contigs from *G*_*k*1_ to generate the combined graph *G*_*k*1–*k*2_, which gives the final set of contigs. Different modules in ScalaDBG are highlighted by different colors.

We unravel ScalaDBG, by describing our protocol for only two distinct *k*-values first, *k*1 and *k*2, and without loss of generality, we assume $k1 < k2$. Figure 2 shows the workflow of ScalaDBG in this scenario.

First, DBGs *G*_*k*1_ and *G*_*k*2_ are built for each *k*-value in parallel. *k*1-mers and *k*2-mers of read set *I* correspond to vertices in *G*_*k*1_ and *G*_*k*2_ respectively. |*G*_*k*1_| and |*G*_*k*2_| represent the number of vertices in *G*_*k*1_ and *G*_*k*2_ respectively. Since $k1 < k2$, Graph *G*_*k*1_ will typically be larger in size, in terms of vertices and edges, *i*.*e*., $|{G}_{k1}| > |{G}_{k2}|$. DBG construction consumes the maximum amount of time in the entire workflow, with the construction of *G*_*k*1_ being the dominant part. Notably, ScalaDBG creates DBGs for the two different *k*-values in parallel, unlike in all prior approaches. Observe that branching in *G*_*k*1_ will be greater than *G*_*k*2_, while the number of gaps or holes will be higher in *G*_*k*2_, relative to *G*_*k*1_. ScalaDBG produces contigs *C*_*k*1_ from *G*_*k*1_ by detecting maximal paths similar to IDBA’s algorithm, but does not yet create *C*_*k*2_ from *G*_*k*2_.

### Patch phase

Graph *G*_*k*2_ has gaps relative to graph *G*_*k*1_, ans hence our fundamental strategy is to *patch* graph *G*_*k*2_, *i*.*e*., fill the gaps in *G*_*k*2_ using the contigs *C*_*k*1_, since they would have the required information. ScalaDBG generates *k*2-mers from *C*_*k*1_, and inserts them as vertices back into *G*_*k*2_. These freshly added vertices could lead to additional edges, if there exists an overlap of a (*k*2 − 1)-mer in the contig set *C*_*k*1_ or the read set. The resulting graph obtained by filling gaps of *G*_*k*2_ using *C*_*k*1_ is represented as *G*_*k*1−*k*2_, and is used for the final contig set generation.

#### Example for ScalaDBG

Consider an example to illustrate the build and patch phases of ScalaDBG with *k*-values of 3 and 4. The graphs *G*_3_ and *G*_4_ are obtained by ${k}_{1}=3$, and ${k}_{2}=4$, and are shown in Fig. 1(a,b). In ScalaDBG, during the build phase, *G*_3_ and *G*_4_ are constructed in parallel. Contig set *C*_3_ obtained from graph *G*_3_ is *{AATGCCGT*, *ACGAA*, *ACGT*, *CGTACG}*. In the patch phase of ScalaDBG, graph *G*_4_ is patched with contigs in *C*_3_. 4-mers obtained from *C*_3_ are *{AATG*, *ATGC*, *TGCC*, *GCCG*, *CCGT*, *ACGA*, *CGAA*, *ACGT*, *CGTA*, *GTAC*, *TACG}*. Of these 4-mers, if two consecutive 4-mers are substrings of a sequence in the contig set, they are inserted as vertices in *G*_4_ and are connected by an edge. Vertices *GTAC* and *TACG* are connected by an edge in *G*_4_ to get *G*_3–4_. *G*_3–4_ is shown in Fig. 1(c). Final contig set *C*_3–4_ generated from *G*_3–4_ is $\{TACGTACG,TACGAA,AATGCCGT\}$.

### Patching multiple *k*-values in parallel

**Figure 3:**
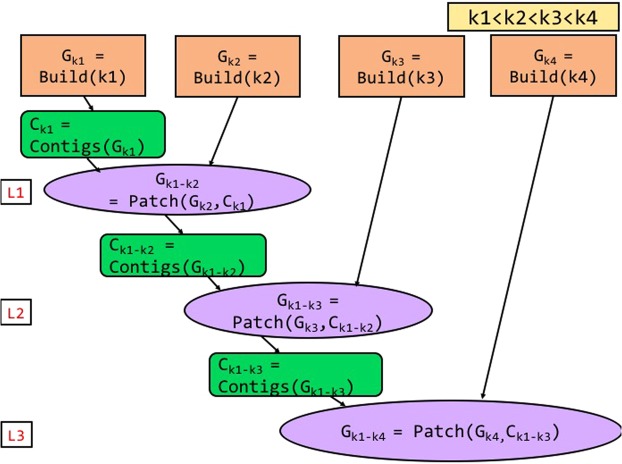
Workflow of ScalaDBG using serial patching, called ScalaDBG-SP for 4 *k*-values, with $$k1 < k2 < $$$$k3 < k4$$.

**Figure 4:**
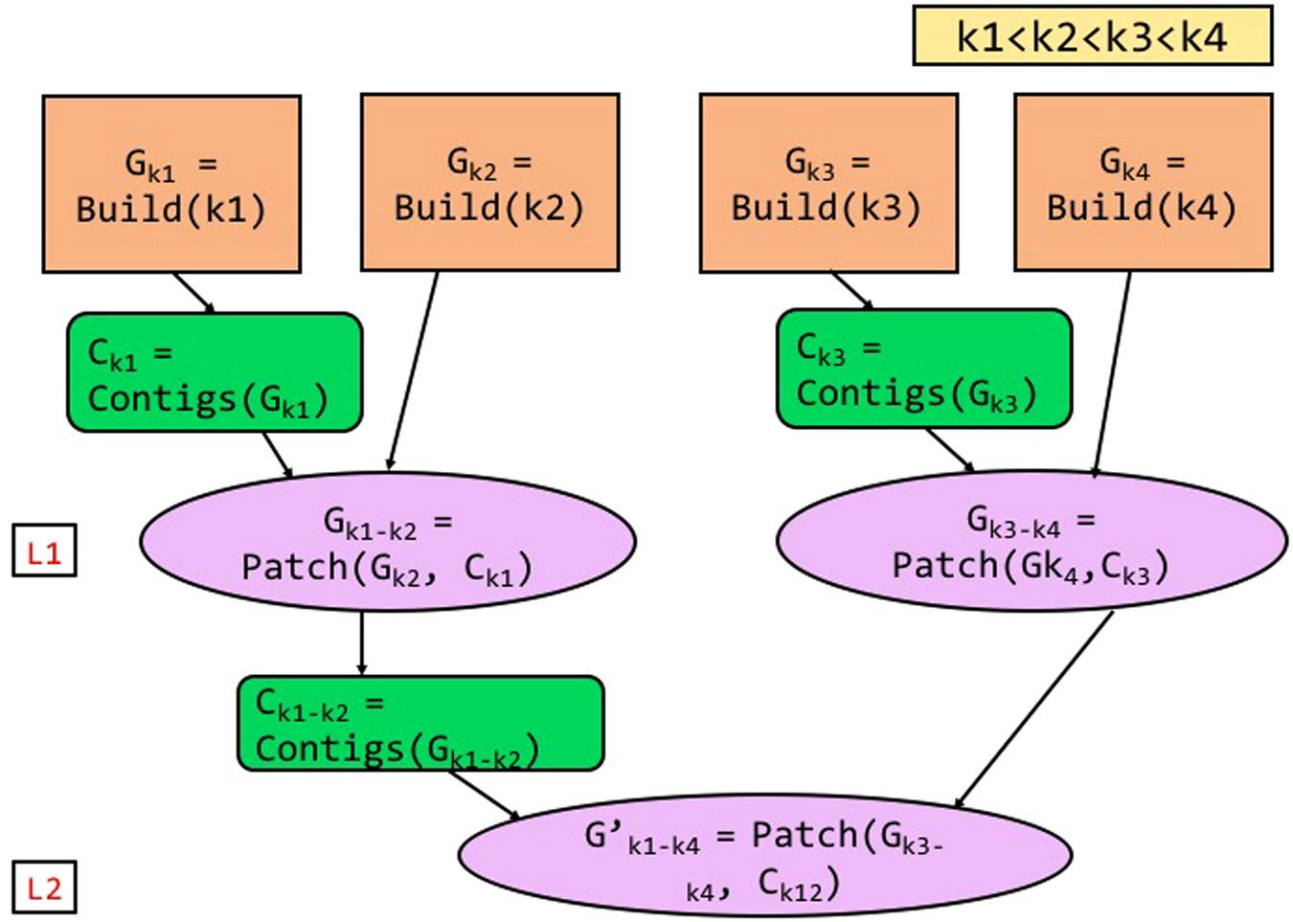
Workflow of ScalaDBG using parallel patching, called ScalaDBG-PP for 4 *k*-values, with $$k1 < k2 < $$$$k3 < k4$$.

ScalaDBG provides two options in the patch phase when the total number of *k*-values used in the DBG creation is greater than 3. Figure 3 shows the serial patching process, and Fig. 4 shows the parallel patching process when there are four different *k*-values: *k*1, *k*2, *k*3, *k*4, and $k1 < k2 < k3 < k4$. As explained earlier, individual DBGs corresponding to each of the *k*-values are constructed in parallel. In serial patching, contigs are first generated for the lowest *k*-valued (*k* = *k*1) graph *G*_*k*1_. They are used to patch the graph corresponding to the next higher *k*-value (*k*2). In this manner, sequentially and repeatedly, contigs generated from a lower *k*-valued patched graph are used to patch the next higher *k*-valued graph. The ultimate set of contigs are obtained from the final patched graph. To summarize, in the serial variant, ScalaDBG performs graph building for individual *k*-values in parallel, but patching and contig generation processes are serial. While this simple approach is easier to implement, unfortunately it reduces the benefits of parallelism since the number of serially executed patch operations grow *linearly* with number of *k*-values. The total serial patch time is a significant portion of the total execution time of ScalaDBG for a long chain of *k*-values. To overcome this problem, ScalaDBG intelligently selects multiple patch operations to be executed in parallel. Rather than adopting an ad-hoc approach to patch graph-pairs, ScalaDBG uses a disciplined policy to patch pairs of adjacent graphs to generate a single graph, since average distance in *k*-values for each DBG is the lowest in this configuration. Prior work has shown that smaller jumps in *k*-values results in better quality aggregated DBGs^6^. Thus, multiple patch operations are performed in parallel akin to a reduction tree model. ScalaDBG’s parallel patching is demonstrated in Fig. 4, where graphs *G*_*k*2_ patched from contigs *C*_*k*1_ of graph *G*_*k*1_, and *G*_*k*4_ patched from contigs *C*_*k*3_ of graph *G*_*k*3_ are processed in parallel. In this manner, ScalaDBG accomplishes both graph building and patching in parallel, and reduces the growth of serialized patching steps to only a *logarithmic* factor of the number of different *k*-values. ScalaDBG achieves higher scope for parallelism when higher number of *k*-values are applied in graph construction. In the remaining paper, we denote ScalaDBG employing serial patching as ScalaDBG-SP (serial-patch ScalaDBG) and ScalaDBG performing parallel patching as ScalaDBG-PP (parallel-patch ScalaDBG). Since ScalaDBG-SP and ScalaDBG-PP merge different pairs of graphs together in the intermediate steps, the final contigs generated by the two methods may differ.

More concretely, here is an example to show why the contigs from serial and parallel patch can vary. Say, we are using 8 *k*-values namely 10, 12, 14, 16, 18, 20, 22, 24, with a step size of 2. In ScalaDBG-SP, the graphs will be patched in order, in this manner: *G*_10–12_, followed by *G*_10–14_, *G*_10–16_ … *G*_10–24_. In other words, the difference between *k*-values associated with adjacent graphs, or step size remains 2, and the contigs generated by the previous graph have lengths close to the next graph. On the other hand, in ScalaDBG-PP, graphs created in stage 1 will be *G*_10–12_, *G*_14–16_, *G*_18–20_, and *G*_22–24_, step size is 2. In stage 2, graphs created will be *G*_10–16_, *G*_18–24_, step size is 4. In stage 3, graph created will be *G*_10–24_, step size is 8. Peng *et al*.^6^ show that a longer contig in the graph obtained with step size *s*, is not present in the graph obtained with step size *s*′, where *s* < *s*′. Since maximum step size in ScalaDBG-PP is higher than in ScalaDBG-SP, (8 > 2), some contigs in ScalaDBG-SP might be absent in ScalaDBG-PP, and the final contig quality in ScalaDBG-PP might drop. Intuitively, the reason that contigs of graphs with close *k*-values should be patched, is that contigs generated from previous *k*-values might not be long enough to connect any nodes in the next graph, when step is large. Say, we are using 4 *k*-values, *k*1, *k*2, *k*3, *k*4 where $k1 < k2 < k3 < k4$ and the minimum distance between any two *k*-values is >=2. In the sequential version, we first build *G*_*k*1_ and get contigs. *G*_*k*1_ could have contigs of length *k*2 + 1, and they could be used to patch *G*_*k*2_. However, they cannot be used to patch graph *G*_*k*3_ since they would not be long enough to connect vertices in graph *G*_*k*3_. In addition, bubble merging and dead-end removal phases prune incorrect vertices and edges in the graphs based on their multiplicity information which could be different in ScalaDBG-SP and ScalaDBG-PP. With deeper trees, the difference in quality of the PP and SP variants is possibly going to be larger and there is a tradeoff between compute efficiency and quality. Empirically we find that the difference is not statistically significant, as shown in Section 6.

### Efficient scheduling of multiple *k*-values

**Figure 5:**
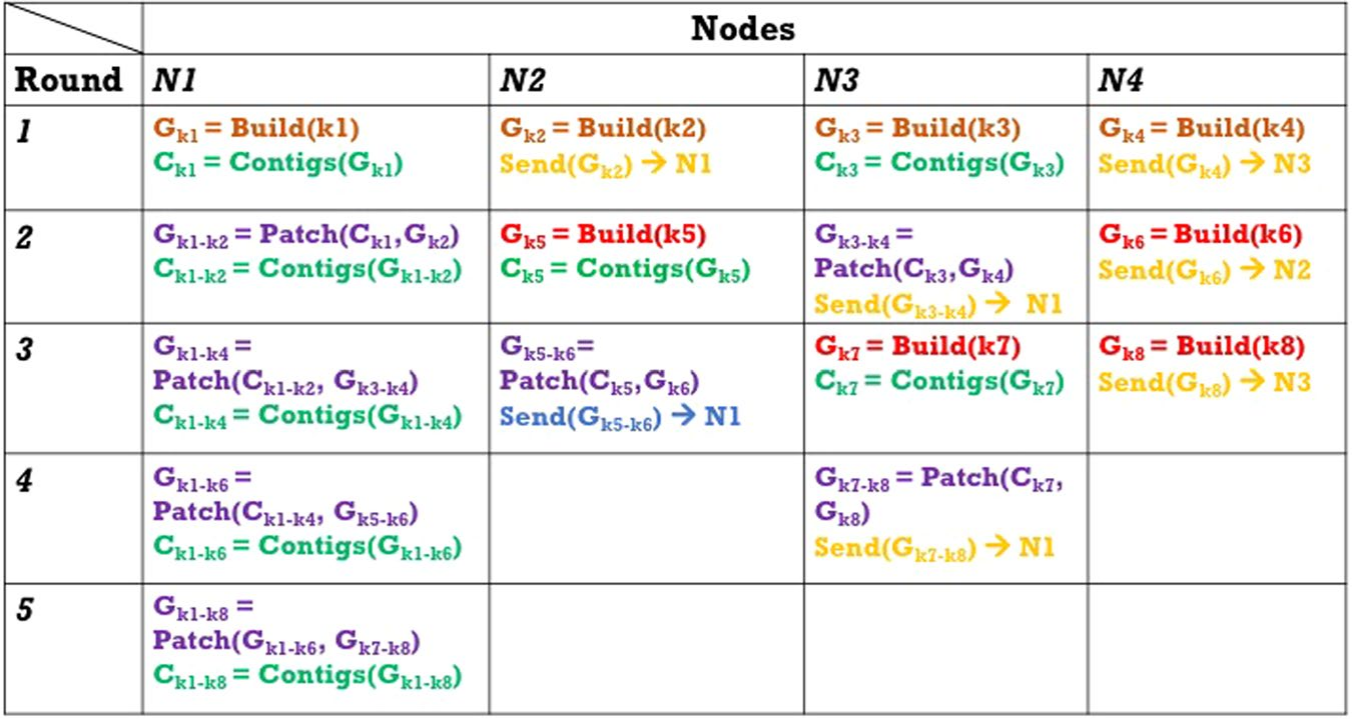
Schedule created by the ScalaDBG Scheduler for 8 *k*-values and 4 nodes. Different computational nodes in the cluster execute different tasks in each round of the workflow.

ScalaDBG’scheduler adopts a greedy strategy to perform assembly for a given number of *k*-values and set of compute nodes, such that utilization of nodes in the cluster is maximized. We discuss the operation of the scheduler for the tree reduction pattern of ScalaDBG-PP (ScalaDBG-SP has a simpler form of a scheduler and is omitted here for space). The generated schedule consists of a set of *rounds* as shown in Fig. 5. Each round is the assignment of a *task* to a node, where a task means the node builds a graph or patches an existing graph with contigs from another graph, or the node is idle. The rounds continue until the final graph is obtained.

The scheduler uses the following observation in creating the schedule: building graph for *k*1 will take longer than building graph for *k*2, where $k1 < k2$ because $|{G}_{k1}| > |{G}_{k2}|$. Hence the node processing *k*2 will get done earlier, and asynchronously send graph *G*_*k*2_ to the node building graph *G*_*k*1_, thus hiding the latency of the communication. This node is then free to take up the next task, either creating a new graph or patching an existing graph. The scheduler consistently overlaps computation with communication in this manner. Similarly, nodes patching higher *k*-valued graphs will finish their tasks earlier than nodes patching lower *k*-valued graphs. Hence these nodes will asynchronously send the patched graphs, and start building the graph for the next *k*-value. The tasks are assigned such that the number of idle nodes in any round is reduced. The amount of work done in each round is not necessarily the same across the nodes, *e*.*g*., in round 1, node 4 does less work than node 3, which does less work than node 2, which does less work that node 1. We use the following example to explain the ScalaDBG scheduler. In this example, there are four available nodes (*n*1, *n*2, *n*3, *n*4) and 8 input *k*-values, *k*1–*k*8, with $k1 < k2 < \ldots  < k7 < k8$. We explain the processing done at each node during each round with the representation shown in Fig. 5. In round 1, all nodes build new graphs from the *k*-values, *k*1–*k*4. Once done with the graph construction, node *n*2 sends graph *G*_*k*2_ to node *n*1 and node *n*4 sends graph *G*_*k*4_ to node *n*3. In round 2, node *n*1 does the patching to generate graph *G*_*k*1–*k*2_ and similarly node *n*3 does a patching while nodes *n*2 and *n*4 build new graphs. This way the different rounds continue till the final graph ${G}_{k1-k8}$ is assembled in node *n*1. Note that in the later rounds, some of the nodes become idle as there are no more tasks to schedule. In this manner, arbitrary number of *k*-values are scheduled to run on a set of nodes by ScalaDBG’s scheduler.

## Methods - Implementation of ScalaDBG

Algorithms 1 and 2 provides the pseduo code listing for ScalaDBG-SP and ScalaDBG-PP respectively.

*n* denotes the total number of *k*-mers used for assembly, *mink* denotes minimum *k*-mer size, *maxk* denotes the maximum *k*-mer size, and *step* represents the increment between each *k*-mer graph built. Each process computes the DBG corresponding to its designated *kmer*_*size* by reading the input read files. In ScalaDBG-SP (Algorithm 1), *N* MPI processes are used to construct the DBGs in parallel, and they then send them to a single process (the Master process) which receives the constructed DBGs. The Master process patches the graphs in sequential order (similar to Fig. 3), to create the final graph. In ScalaDBG-PP (Algorithm 2), after constructing the individual DBGs, the process are divided into two roles - either send a DBG or recieve a DBG. This role division is done in an equitable fashion. The receiving process then completes the patching of the DBG. This process is then used further for contig generation. For the subsequent stages, this process of role division and contig generation is repeated, until only one process receives the final graph. MPI implementation is done in such a way, that this process is always the Master process, and it applies the final patching and generates the ultimate contigs.

## Discussion: Correctness, Implications, and Generality of ScalaDBG

In this Section, we first establish the equivalence of graphs obtained by the build and serial patching processes of ScalaDBG and the iterative build process of IDBA-UD. We then discuss the implications of the parallel patching and the graph-simplification procedures on the output of ScalaDBG.

### Correctness of the ScalaDBG Methodology (essentially showing the equivalence between the final graphs of ScalaDBG-SP and iterative IDBA-UD)

For a fixed iteration set of *k*-values, starting from *k* = *kmin* to *k* = *kmax*, the final graphs obtained by ScalaDBG-SP and IDBA-UD are identical. We request the reader to refer to the proof of Theorem 4.1 in our previous work^10^. The proof uses Mathematical Induction to establish the equivalence between the final graphs using the two methods, namely: ScalaDBG-SP and iterative IDBA-UD.

**Algorithm 1:**
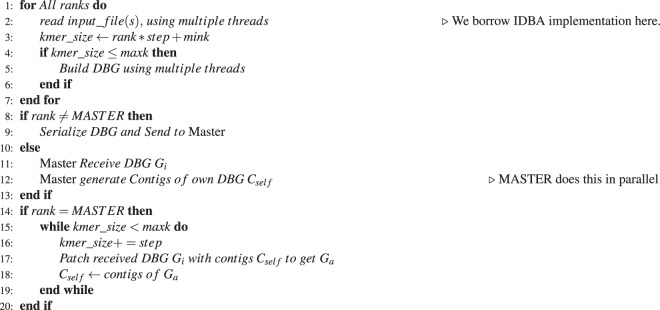
ScalaDBG with serial patching. *N* MPI processes have ranks (identifiers) from 0 to *N* − 1. MPI process with rank 0 is referred to as the Master process.

**Algorithm 2:**
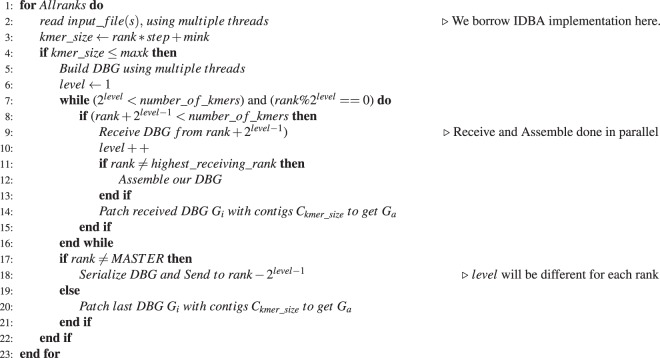
ScalaDBG with parallel patching. *N* MPI processes have ranks (identifiers) from 0 to *N* − 1. MPI process with rank 0 is referred to as the Master process.

### Implications of ScalaDBG’s methods

While the build and patch processes for iterative IDBA-UD and ScalaDBG are the same, the assembly metrics for the two variants of ScalaDBG and IDBA-UD are different. This can be attributed to two processes:

- Out-of-order patching process for ScalaDBG –PP
- Graph simplification processes in IDBA-UD, such as dead-end removal (to prune incorrect vertices and edges based on their multiplicity information and path length) and bubble merging (merging paths of similar length with the same start and end vertex). In IDBA-UD, the graph simplification process is applied to each graph during the build and after traversal, while in ScalaDBG, it is only applied after each patching.

ScalaDBG-PP builds graphs in a different order (than ScalaDBG-SP and IDBA-UD) when the number of *k*-values is greater than 3. IDBA-UD sequentially builds graphs where the *k*-value used in the next iteration differs from the *k*-value used in the previous iteration by *step*-*size*. ScalaDBG-PP performs a pair-wise reduction to patch graphs at every level. Lower-depths in the parallel reduction tree have graphs with *k*-value difference greater than *step*-*size*. Hence, the order of patching graphs is different in ScalaDBG-PP and IDBA-UD.

In addition, for the graphs built by ScalaDBG and IDBA-UD, vertices and edges have different multiplicity information. Hence, the graph-simplification procedures generate different contig sets with different assembly metrics for ScalaDBG-SP, ScalaDBG-PP and IDBA-UD. However, in our evaluation section, we will show that the difference is not statistically significant.

### Generality of ScalaDBG’s methods

**Figure 6:**
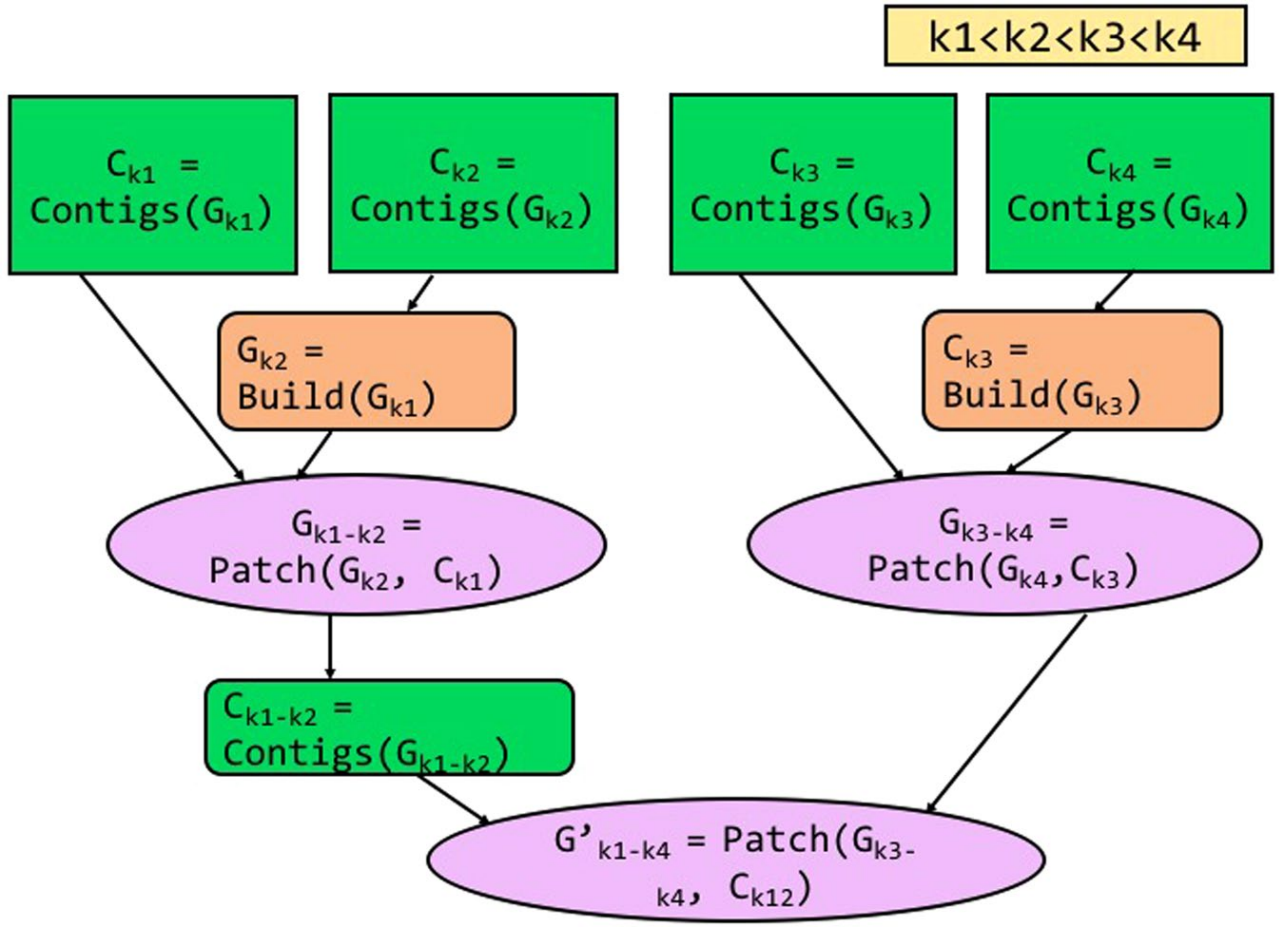
General assembler used in conjunction with ScalaDBG’s technique. Contigs C_k1_, C_k2_, C_k3_, and C_k4_ are obtained using a general assembler.

We demonstrate how we can leverage an out-of-the-box DBG-based assembler and parallelize DBG construction for a chain of *k*-values. ScalaDBG modularizes different stages in assembly such as building the graph, patching the graph with contig sets, and the generation of contigs. Hence, these modules can be *reused* to combine the contigs of a single *k*-value assembler, such as Velvet. We use an example to explain this application, as shown in Fig. 6. There are four different *k*-values, in increasing order: *k*1, *k*2, *k*3, and *k*4. Initially, a single *k*-value assembler is run for each of these *k*-values in parallel to generate the contig sets. The obtained contig sets are used to patch the graph associated with the contig set of the next higher *k*-value (*k*2). Graphs *G*_*k*2_ and *G*_*k*4_ are built from the contig sets *C*_*k*2_ and *C*_*k*4_, respectively, according to the definition of DBG, considering the contig set as the input read set. Then, the graphs *G*_*k*2_ and *G*_*k*4_ are patched using contig sets *C*_*k*1_ and *C*_*k*3_, respectively. After this stage, the method follows the standard parallel-patch workflow of ScalaDBG. Note that we can also employ serial patching to get the final contig set. (*i*.*e*., ScalaDBG-SP).

## Evaluation and Results

### Evaluation setup and datasets

We used an Intel Xeon Infiniband cluster for our experiments, with each node having Intel Xeon E5-2670, 2.6 GHz, with 16 cores per node and 32 GB of memory and the nodes connected with QDR Infiniband. We used the latest version of IDBA-UD (1.1.1)^7^. The datasets are enlisted in Table 1. The *S*. *aureus* and SAR 324 single-cell datasets are obtained from^22^ and the CAMI benchmark datasets comprise our metagenomics datasets^23^. For ScalaDBG, the number of nodes were equal to the number of *k*-values while IDBA-UD can only run on a single node. Existing scaffolding techniques can be applied to output contigs that are obtained from ScalaDBG to get the final assembly. We only focus on the outputs at the contig generation stage rather than after the scaffolding process because ScalaDBG’s novelty in this implementation is restricted to the contig generation process.

### Relevance of datasets

A single cell is the ultimate denomination in a multicellular organism. For example, the human body consists of roughly 37.2 trillion cells living in harmony. However, in cancer, this harmonious equilibrium is lost and this is where even one single cell can wreak havoc by evolving into a malignant tumor mass, wherein the lineages diverge and form distinct populations giving rise to what is known as clonal diversity. While in the past, technological limitations required micrograms of input tissue mass resulting in an *average* signal being emanated from a complex mass of heterogeneous cell types, single-cell sequencing (SCS) methods can now revolutionize the understanding of cancer biology, affording insights into the role of rare cells in the evolution of cancer. In the case of metagenomic and single cell sequencing datasets, sequencing depths of different regions of a genome, or genomes from different organisms are exceedingly uneven. Hence multiple *k*-values are required for accurately assembling the datasets. So we evaluate ScalaDBG and IDBA-UD using these relevant datasets.

### Performance tests

**Figure 7:**
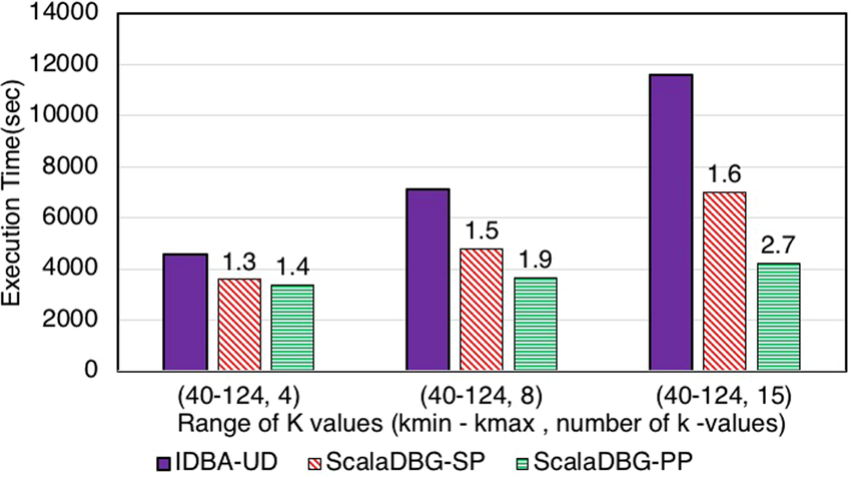
Execution Time comparison for IDBA-UD, ScalaDBG-SP, ScalaDBG-PP on RM1 dataset. ScalaDBG runs on a cluster with the number of nodes being equal to the number of *k*-values.

**Figure 8:**
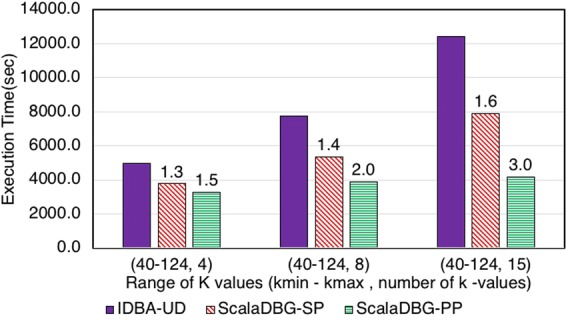
Execution Time comparison for IDBA-UD, ScalaDBG-SP, ScalaDBG-PP on RM2 dataset.

**Figure 9:**
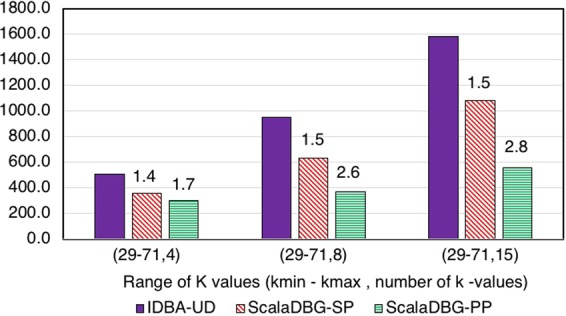
Execution Time comparison for IDBA-UD, ScalaDBG-SP, ScalaDBG-PP on the SC-*E*. *coli* dataset. Speed up w.r.t. IDBA-UD running on the same *k* value configuration is shown.

**Figure 10:**
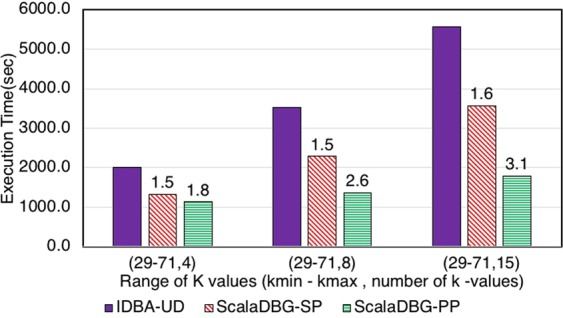
Execution Time comparison for IDBA-UD, ScalaDBG-SP, ScalaDBG-PP on the SC-*S*. *aureus* dataset.

**Figure 11:**
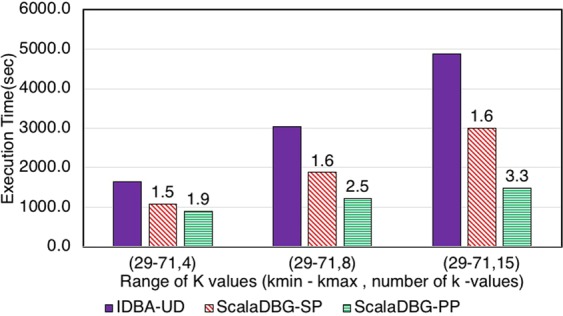
Execution Time comparison for IDBA-UD, ScalaDBG-SP, ScalaDBG-PP on the SC-SAR324 dataset.

**Figure 12:**
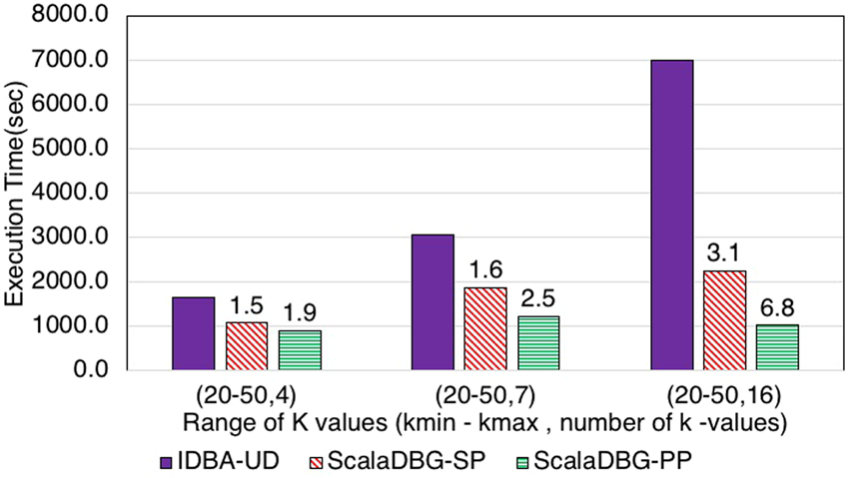
Execution Time comparison for IDBA-UD, ScalaDBG-SP, ScalaDBG-PP on the SC-SAR324 dataset for range (20–50).

Figures 7, 8, 9, 10, 11 and 12 compare the execution time of IDBA, ScalaDBG-SP, and ScalaDBG-PP to generate contigs for the read sets mentioned in Table 1. We generated different *k*-value configurations by varying the number, range and step sizes. For the metagenomics dataset, distinct step sizes of 28, 12, and 6 in the 40–124 range generated 3 different configurations. Similarly, step sizes of 14, 6, and 3 for the 29–71 range generated 3 different configurations for the single-cell sequencing datasets. The single-cell datasets have a narrower range because of the shorter length of the sequenced reads. To vary the number of *k*-values, we used step sizes of 10, 5, and 2 in the range of 20–50 for the SAR324 dataset to obtain 4, 7, and 16 *k*-values, respectively.

These different *k*-value configurations are meant to evaluate the effect on quality and execution time of assembly for ScalaDBG and IDBA-UD. ScalaDBG is deployed on a cluster with number of nodes equal to the number of distinct *k*-values in an experiment, while IDBA-UD can run only a single node. Speedup over IDBA and assembly quality of ScalaDBG increases with increase in *k*-values for all the read datasets. In fact, speedup of ScalaDBG completely depends on the corresponding value of *k*. For example, for the SAR324 dataset, with the *k*-value range between 20 and 50 and a step size of 2, speedup of ScalaDBG-PP is 6.8X while the speedup of ScalaDBG-SP is 3.1X relative to IDBA-UD. Of all the remaining read sets and configurations, ScalaDBG-PP achieves a maximum speedup of 3.3X for the SC-SAR324 readset in the {29–71}, with a step size of 3. ScalaDBG-SP achieves a maximum speedup of 1.6X for RM1, RM2, SC-*S*. *aureus*, and SC-SAR324 readsets in the configurations processing 15k values. For all the datasets and configurations, ScalaDBG is faster than IDBA-UD. Further, speedup is higher for the larger read datasets of RM1, RM2, SAR 324, and *S*. *aureus*. Finally, as would be expected ScalaDBG-PP is always faster than the serial version.

### Accuracy

Tables 2, 3 and 4 show the quality metrics for assembling the datasets in Table 1 using ScalaDBG. We used the QUAST tool^24^ to compare the assemblies obtained using ScalaDBG-SP, ScalaDBG-PP, and IDBA-UD. For the metagenomic datasets, Table 2 reports number of contigs, N50, and max contig length, since the reference assemblies contained multiple genomes instead of a single genome. For SAR324, we did not have access to the reference genome, so Table 4 denotes the coverage, NGA50, and number of misassemblies as NA. Table 3 reports both N50 and NGA50 since we had a single reference genome for the datasets. The most common metric to assess assembly quality is *N50*. N50 is defined as the length of the smallest contig above which 50% of an assembly would be represented (or smallest scaffold if it is applied after scaffold construction), a higher N50 indicating improved assembly. In presence of the reference genome, NGA50 provides more insights into the assembly quality. NGA50 is defined as the contig length such that using equal or longer length contigs that have been aligned to the reference produces 50% of the length of the reference genome; again, a higher NGA50 indicates an improved assembly.

**Table 2:** Accuracy Comparison for Assembler Performance for datasets RM1 and RM2., for specified set of k-values and step size configurations.

Assembler	# Contigs	N50 (bp)	Max Contig Length	# Contigs	N50 (bp)	Max Contig Length
	**RM1 k** = **40**–**124**,**4**			**RM2 k** = **40**–**124**,**4**		
IDBA-UD	96291	8255	641885	123807	2251	572031
ScalaDBG-SP	94958	8104	641902	122427	2281	571953
ScalaDBG-PP	95519	7629	497722	123037	2249	444176
	**RM1 k** = **40**–**124**,**8**			**RM2 k** = **40**–**124**,**8**		
IDBA-UD	95633	10729	772713	121911	2457	563546
ScalaDBG-SP	96849	10183	772927	121955	2582	573903
ScalaDBG-PP	98018	7962	497730	121772	2408	444517
	**RM1 k** = **40**–**124**,**15**			**RM2 k** = **40**–**124**,**15**		
IDBA-UD	95640	11453	772928	121720	2504	563546
ScalaDBG-SP	99857	10182	641918	119814	2679	573903
ScalaDBG-PP	99951	7906	641988	121568	2472	444518

**Table 3:** Accuracy Comparison for Assembler Performance for SC-*E*. *coli* and SC-*S*. *aureus* datasets., for specified set of k-values and step size configurations.

Assembler	# Contigs	N50 (bp)	Max Contig Length	Coverage	NGA50 (bp)	# misassemblies
	**SC**-***E***. ***coli*** **k** = **40**–**124**,**4**					
IDBA-UD	504	41996	133040	93.004	41009	4
ScalaDBG-SP	506	43834	133040	93.086	41309	4
ScalaDBG-PP	333	46016	140917	93.072	41996	3
	**SC**-***E***. ***coli*** **k** = **40**–**124**,**8**					
IDBA-UD	503	42834	140971	93.045	41309	4
ScalaDBG-SP	504	43834	133040	93.064	41996	3
ScalaDBG-PP	333	46016	140917	93.086	42289	4
	**SC**-***E***. ***coli*** **k** = **40**–**124**,**15**					
IDBA-UD	507	42289	133040	93.101	41009	6
ScalaDBG-SP	512	46016	140971	93.093	42289	6
ScalaDBG-PP	333	46016	140917	93.078	42289	4
	**SC**-***S***. ***aureus*** **k** = **40**–**124**,**4**					
IDBA-UD	400	24855	126604	98.121	26379	3
ScalaDBG-SP	377	24855	126604	98.189	26379	3
ScalaDBG-PP	370	24855	126604	98.201	26379	3
	**SC**-***S***. ***aureus*** **k** = **40**–**124**,**8**					
IDBA-UD	412	24855	126604	98.081	26379	3
ScalaDBG-SP	384	24855	126604	98.176	26379	3
ScalaDBG-PP	373	24855	126604	98.205	26379	3
	**SC**-***S***. ***aureus*** **k** = **40**–**124**,**15**					
IDBA-UD	413	24855	126604	98.068	26379	3
ScalaDBG-SP	393	24855	126604	98.167	26379	3
ScalaDBG-PP	374	24855	126604	98.205	26379	3

**Table 4:** Accuracy Comparison for Performance Tests on SC-SAR 324 datasets, for specified set of k-values and step size configurations.

Assembler	# Contigs	N50 (bp)	Max Contig Length	Coverage	NGA50 (bp)	# misassemblies
	**SC**-**SAR324 k** = **29**–**71**,**4**					
IDBA-UD	733	61419	202281	NA	NA	NA
ScalaDBG-SP	709	64747	202281	NA	NA	NA
ScalaDBG-PP	705	62374	202281	NA	NA	NA
	**SC**-**SAR324 k** = **29**–**71**,**8**					
IDBA-UD	742	60700	202281	NA	NA	NA
ScalaDBG-SP	710	64747	202281	NA	NA	NA
ScalaDBG-PP	703	63904	202281	NA	NA	NA
	**SC**-**SAR324 k** = **29**–**71**,**15**					
IDBA-UD	747	60700	202281	NA	NA	NA
ScalaDBG-SP	723	64747	202281	NA	NA	NA
ScalaDBG-PP	712	64795	202281	NA	NA	NA
	**SC**-**SAR324 k** = **20**–**50**,**4**					
IDBA-UD	1082	32119	131087	NA	NA	NA
ScalaDBG-SP	1085	38257	131546	NA	NA	NA
ScalaDBG-PP	1080	38257	131546	NA	NA	NA
	**SC**-**SAR324 k** = **20**–**50**,**7**					
IDBA-UD	1088	33192	131087	NA	NA	NA
ScalaDBG-SP	1087	38257	131546	NA	NA	NA
ScalaDBG-PP	1078	38257	131546	NA	NA	NA
	**SC**-**SAR324 k** = **20**–**50**,**16**					
IDBA-UD	7740	22977	131087	NA	NA	NA
ScalaDBG-SP	8342	24254	131041	NA	NA	NA
ScalaDBG-PP	8118	24254	131041	NA	NA	NA

The table entries reveal that ScalaDBG and IDBA have comparable accuracy metrics in all cases. While the actual numbers for ScalaDBG-SP, ScalaDBG-PP, and IDBA-UD differ due to out-of-order patching and graph simplification process, we confirmed using the *t*-test that these differences are not statistically significant.

### Time distribution for phases of ScalaDBG

**Figure 13:**
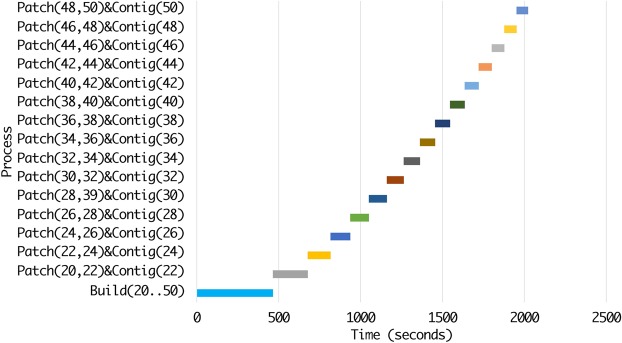
Execution time breakdown of ScalaDBG-SP for SAR 324 dataset, *k*-value range{20–50}, step size 2.

**Figure 14:**
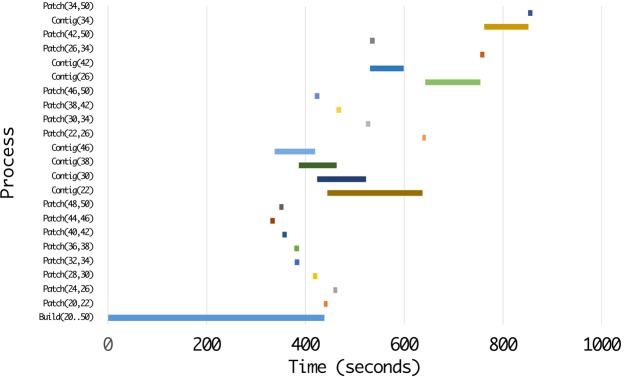
Execution time breakdown of ScalaDBG-PP for SAR 324 dataset, *k*-value range {20–50}, step size 2.

ScalaDBG’s time is spent executing three major tasks: (1) the DBG construction (Build), (2) graph patching (Patch), and (3) contig generation (Contig). We profiled ScalaDBG to determine the contribution of each function to the total execution time of ScalaDBG. We present here the results of assembling the SAR 324 dataset for the *k*-value range of {20–50}, with a step size of 2. The experiment was run on a 16-node cluster. Figures 13 and 14 show the different sub-tasks within each task for ScalaDBG on the Y-axis while the X-axis shows the actual execution time in seconds. Note that in ScalaDBG-SP (Fig. 13), only the DBG construction executes in parallel, while in ScalaDBG-PP (Fig. 14), in addition to build, the patch and contig-generation phases execute in parallel as well. Patch tasks take less time compared to the contig-generation tasks.

As seen in Figs 13 and 14, ScalaDBG-PP overlaps the execution of certain graph-construction, patching, and contig-generation tasks, while in ScalaDBG-SP, all the patching and contig generation tasks are serialized. This detailed profile can be used to optimize ScalaDBG further.

Unlike IDBA-UD, ScalaDBG-SP and ScalaDBG-PP do not update the input read set at each iteration. Instead, each graph construction in ScalaDBG starts with the original read set. IDBA-UD updates the read set at each iteration, scanning all reads in the read set, and removing the ones that are already contained in any of the contigs in the contig set. However, if the reduction in the input read set is not significant at each iteration, then the overhead of updating the read set for IDBA-UD starts to dominate. Especially for lower *k*-values, excessive branching can lead to less reduction in the read set, increasing the overhead for IDBA-UD. In addition, the patch and contig generation occurs only logarithmic number of times in ScalaDBG-PP as compared to IDBA-UD and ScalaDBG-SP.

If we serialize the execution time of the parallel processes in ScalaDBG-SP and ScalaDBG-PP, shown in Figs 13 and 14, the serial execution time for ScalaDBG-SP is 6455 seconds and for ScalaDBG-PP is 6457 seconds. The execution time for IDBA-UD is 6897 seconds. Out of this total time, 86% of the overall execution time can be parallelized. Hence, the maximum speedup for ScalaDBG-PP is 6.8X. ScalaDBG-SP performs the patch and contig generation serially, hence its speedup drops to 3.1X. For this dataset and *k*-value range configuration, the additional work done by ScalaDBG is offset by the work done by IDBA-UD in updating of the read set.

### Comparison with the distributed assembler ABySS

ABySS^3^ is a distributed assembler and it parallelize the execution of DBG construction for a single *k*-value on multiple nodes using MPI. Hence, we compared distributed ScalaDBG and state-of-the-art distributed assembler ABySS. We measured the execution time and quality of ScalaDBG and ABySS for completing the assembly of SC-SAR-324 dataset.

Both, ScalaDBG and ABySS were deployed on a cluster of 4 nodes, and could utilize all cores on the nodes. ScalaDBG was input *k*-value range of 20–50, with a step size of 10, while ABySS was executed using a median value of *k* = 35 to present a fair quality comparison. As shown in Table 5, ScalaDBG is significantly faster (2.5X) and produces better quality assembly than ABySS. Since ScalaDBG uses multiple *k*-values, namely, 20, 30, 40, and 50, as opposed to ABySS that just uses a single *k*-value of 35, ScalaDBG has higher N50 and maximum-contig length as compared to ABySS.

**Table 5:** Accuracy and Performance comparison on SC-*SAR 324* datasets for ScalaDBG-PP and ABySS. ScalaDBG-PP has higher accuracy and is significantly faster than ABySS.

Assembler	Execution Time (sec)	N50 (bp)	Max Contig Length
Abyss	2240	37486	131365
ScalaDBG	892	38257	131546

### Scalability tests

**Figure 15:**
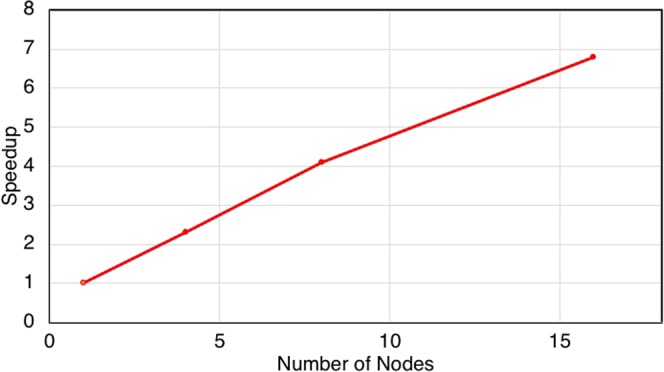
Speedup for ScalaDBG assembling SAR 324 dataset *k*-value range {20–50}, step size 2, speedup shown w.r.t. ScalaDBG running on 1 node.

To evaluate the scaling out for ScalaDBG, we used the SAR324 dataset. We varied ScalaDBG’s *k*-values, ranging from {20–50}, with a step size of 2, which translates to 16 *k*-values. The speedup is measured when scaling from 1 to 16 nodes, as shown in Fig. 15, resulting in a 6.8X speedup in relation to the baseline when running on a single node. ScalaDBG scales at nearly constant efficiency, as judged from the slope of the speedup curve. The speedup demonstrates that ScalaDBG can scale out efficiently on a multi-core and multi-node cluster.

## Conclusion

The rapid progress of sequencing instruments and algorithms have resulted in the need for faster and more efficient assembly algorithms. Further, advances in single-cell sequencing and metagenomics domains for assessing cancer heterogeneity and the microbiome, respectively, are hindered by the time needed for genome assembly. Existing iterative DBG assemblers, such as IDBA-UD, generate longer contigs at the cost of significantly longer graph construction times due to the serial construction process for a set of *k*-values. In ScalaDBG, we break the serial graph construction process into multiple parallel processes. Further, our technique is also extensible in that it can be applied to other DBG-based assemblers.

## Data Availability

The datasets analyzed during the current study are available at the following links: http://bix.ucsd.edu/projects/singlecell/nbt_data.html https://data.cami-challenge.org (Critical assessment of metagenome interpretation–a benchmark of computational metagenomics software).
